# A Special Issue on Marek’s Disease Virus—The Editors’ View

**DOI:** 10.3390/microorganisms11030805

**Published:** 2023-03-21

**Authors:** Benedikt B. Kaufer, Mark S. Parcells, Luca D. Bertzbach

**Affiliations:** 1 Institute of Virology, Freie Universität Berlin, 14163 Berlin, Germany; 2 Veterinary Centre for Resistance Research (TZR), Freie Universität Berlin, 14163 Berlin, Germany; 3 Department of Animal and Food Sciences, University of Delaware, Newark, DE 19716, USA; 4 Department of Viral Transformation, Leibniz Institute of Virology (LIV), 20251 Hamburg, Germany; luca.bertzbach@leibniz-liv.de

Marek’s disease virus (MDV), an Alphaherpesvirus belonging to the genus Mardivirus, causes T cell lymphomas in chickens and remains one of the greatest threats to poultry production worldwide. While losses caused by Marek’s disease have been reduced through live-attenuated vaccines, field strains have increased in virulence over recent decades. MDV research has led to a profound understanding of virus-induced pathogenesis and tumor development [1,2,3]. Our goal with this Microorganisms Special Issue on Marek’s disease virus was to collect manuscripts that would provide deeper insights into MDV infection, lytic replication, and latency in vitro and in vivo. Moreover, we assembled reports that provide novel data on pathogenesis, immune system interactions, as well as state-of-the-art concepts to identify approaches to control MDV infections. We were happy to edit seven research articles, three short communications, and a review article on these diverse aspects of MDV infections.

A timely summary of the major methods of manipulating herpesvirus genomes was provided by Liao et al., with an emphasis on their applications in MDV research. The authors reviewed both the “traditional” methods, such as site-directed mutagenesis and the use of cosmids, and more recent methods, like bacterial artificial chromosomes (BACs) and CRISPR/Cas9-mediated genome editing [4]. The same laboratory contributed two additional articles that dealt with the MDV-encoded US3 protein kinase and how this protein regulates viral replication, as well as the viral manipulation of cellular PML nuclear bodies [5,6].

You et al. reported the characterization of a new splice variant of viral interleukin 8 (vIL-8), a chemotactic cytokine, showing that this new vIL-8 isoform is dispensable for MDV replication and pathogenesis [7].

Two papers addressed pressing questions in MDV research using CRISPR/Cas9 approaches: the switch between lytic and latent MDV infections and the use of gene technology to generate MDV-resistant chickens. Roy et al. used CRISPR activation (CRISPRa) of viral genes to investigate the lytic–latent switch. This switch seems to rely on the two MDV phosphoproteins pp24 and pp38 [8]. Challagulla et al., on the other hand, generated and challenged transgenic chickens that constitutively express Cas9 and MDV-specific guide RNAs, aiming to increase virus resistance in these birds [9].

In an attempt to improve MDV vaccines in the context of immunosuppression commonly caused by MDV and its widely used vaccines, Conrad et al. generated genetically modified vaccine candidates and assessed their vaccine-mediated protection and immunosuppressive effects [10].

Three contributions highlight the value of MDV infections in chickens as a model for human diseases. Erf et al. used a Mardivirus in vivo model to gain new insights into vitiligo, a chronic autoimmune disorder [11]. A genomics study by Steep et al. revealed that key somatic mutations in the *Ikaros* cancer driver gene can contribute to MDV-induced tumorigenesis. Similar mutations also exist in some human cancers, suggesting that somatic mutations may explain the origin of MDV-induced lymphomas [12]. Gennart and Petit et al. investigated the role of a cellular micro RNA, miR-126, in MDV infection. Interestingly, miR-126 is known to play key roles in human cancers as well [13].

Finally, the methods paper on a cell culture system to study MDV integration by You and Vychodil et al. provided a method that allows the investigation of molecular mechanisms of MDV telomere integration, as well as the quantification of virus genome integration, without requiring laboratory animals [14].

Although much progress has been made in MDV research and a growing body of knowledge about this DNA tumor virus exists, a number of challenges need to be overcome. Through this Special Issue, we hope to contribute to a better understanding of MDV as an important poultry pathogen, but also as a valuable animal model for herpesvirus research, virus–host interactions, and virus-induced tumorigenesis.
